# Volunteer geographic information in the Global South: barriers to local implementation of mapping projects across Africa

**DOI:** 10.1007/s10708-020-10184-6

**Published:** 2020-04-08

**Authors:** Jason C. Young, Renee Lynch, Stanley Boakye-Achampong, Chris Jowaisas, Joel Sam, Bree Norlander

**Affiliations:** 1 Technology and Social Change (TASCHA), University of Washington, Seattle, WA USA; 2 African Library and Information Associations and Institutions, Accra, Ghana

**Keywords:** VGI, Global South, Crowdsourcing, Public libraries, Mapping

## Abstract

The world is awash in data—by 2020 it is expected that there will be approximately 40 trillion gigabytes of data in existence, with that number doubling every 2 to 3 years. However, data production is not equal in all places—the global data landscape remains heavily concentrated on English-speaking, urban, and relatively affluent locations within the Global North. This inequality can contribute to new forms of digital and data colonialism. One partial solution to these issues may come in the form of crowdsourcing and volunteer geographic information (VGI), which allow Global South populations to produce their own data. Despite initial optimism about these approaches, many challenges and research gaps remain in understanding the opportunities and barriers that organizations endemic to the Global South face in carrying out their own sustainable crowdsourcing projects. What opportunities and barriers do these endemic organizations face when trying to carry out mapping projects driven by their own goals and desires? This paper contributes answers to this question by examining a VGI project that is currently mapping public libraries across the African continent. Our findings highlight how dramatically digital divides can bias crowdsourcing results; the importance of local cultural views in influencing participation in crowdsourcing; and the continued importance of traditional, authoritative organizations for crowdsourcing. These findings offer important lessons for researchers and organizations attempting to develop their own VGI projects in the Global South.

## Introduction

The world is awash in data. By 2020 it is expected that there will be approximately 40 trillion gigabytes (40 zettabytes) of data in existence, with that number doubling every 2 to 3 years (Petrov 2019). For context, Internet users currently share more than 500,000 photos on Snapchat, watch over 4,000,000 videos on YouTube, and send more than 450,000 tweets *every minute* (Marr 2018). Amongst researchers there is broad consensus that this explosion of digital data, along with increased accessibility of digital information and communication technologies (ICTs), has had a profound effect on political, economic, and social processes across the globe (Benkler 2006; Bimber 2007; Castells 2004). However, neither data production nor the presence of the digital technologies and networks that support them is equal in all places around the world (Castells 2004). The global data landscape remains heavily concentrated on English-speaking, urban, and relatively affluent locations within the Global North (Burns 2014; Caquard 2014; Graham and Zook 2013; Young 2019a, b).

Nor is it simply that the datasets most richly and accurately represent locations in the Global North—residents in the North also tend to have the highest ability to *control* how data are produced, owned, analyzed, and shared. This issue of control is perhaps more problematic than the current lack of data, given that data are increasingly emerging from the Global South with attendant interest in leveraging them for economic change, development, and governance (Kshetri 2014; Mann 2017; Taylor 2017; Taylor and Schroeder 2014). Scholars are now calling attention to the emergence of new forms of digital and data colonialism. This research describes both how denizens of the Global South are exploited as data producers to reproduce neoliberal political economies (Ettlinger 2016; Thatcher et al. 2016) and also how existing datasets are used to extend Western cultural hegemonies and development visions to new locations (Burns^2014^; Taylor and Broeders 2015; Young 2019a). Unfortunately, there remains widespread agreement that this body of work remains underdeveloped, and much of the current scholarship has focused on critique rather than solutions (Dé et al. 2018).

One partial solution to these issues may come in the form of crowdsourcing. In an ideal world crowdsourcing projects would allow individuals and organizations in the Global South to produce their own data and establish control over what happens to the data that they produce. At times organizations and researchers are too optimistic in their descriptions of crowdsourcing as a ‘panacea’ for problems afflicting the Global South (e.g., Bott and Young 2012), but even those that take a more critical approach see great promise. Lievano (2017), for example, argues that crowdsourcing makes *more* sense in the Global South given current data gaps, and Ingwe (2017) argues for the increased adoption of the method by civil society organizations across sub-Saharan Africa. Already many map-based crowdsourcing, also known as volunteer geographic information (VGI), projects have been successfully carried out across the continent (Yilma 2019).

Despite this work, many challenges and research gaps remain. There is a paucity of data available within many African countries, and VGI is largely driven by international non-governmental organizations (NGOs) instead of African organizations (Omanga and Mainye 2019). As a result, there is a risk that these projects feed back into the data inequalities and digital colonialism that VGI would ideally resist. Needed are more discussions of how to build the capacity of African organizations and governments to institute and sustain their own comprehensive and broadly implemented VGI programs. What opportunities and barriers do these endemic organizations face when trying to carry out mapping projects driven by their own goals and desires? This paper contributes answers to this question by examining a VGI project that is currently mapping public libraries across the African continent. This project is a collaboration between researchers at the University of Washington Information School and practitioners at the African Library & Information Associations and Institutions (AfLIA), and a primary goal of the work is to build the capacity of AfLIA to sustainably collect VGI over the long run. Recently the project team held a stakeholders’ meeting with representatives from 22 different countries, each of whom is coordinating the mapping effort within their respective country. This paper analyzes their feedback on the challenges and opportunities they have faced in implementing the project in their countries, as well as their thoughts on the long-term sustainability of data collection. Their lessons can help to inform other large-scale data collection efforts in the Global South.

## Volunteer geographic information in the Global South

VGI projects like the one described in this paper have been made possible by fundamental shifts in processes and understandings of geospatial knowledge production, enabled by mobile and Web 2.0 technologies. In this section we describe the emergence of VGI, as well as the related concepts of crowdsourcing, user generated content (UGC), and neogeography. We also review current efforts to extend VGI to countries and communities across the Global South. We argue that there is insufficient literature describing the challenges and opportunities that African organizations face in implementing and sustaining their own VGI projects.

Crowdsourcing, and the related processes described in this section, have been enabled by the development and extension of digital networks which greatly augment the human capacity for information storage, analysis, and communication (Castells 2004). The digital technologies that access these networks are now cheaper than ever, are relatively ubiquitous, and relatively easy to use—meaning that more individuals now have more opportunities to produce, communicate, and use digital media than ever before (Benkler 2006; Bimber 2007; Castells 2004). Furthermore, the digital reach of these networks allow individuals to access global networks of other users, giving them the ability to connect to other users across very large distances. This produces a small-world effect that allows information to travel quickly and widely within those networks to reach broad audiences (Barabasi and Bonabeau 2003; Bennett and Segerberg 2013; Buchanan 2002; Lotan et al. 2011). These technologies thus allow individuals across vast scales to interact and collaborate with one another to co-produce knowledge, in ways that only governments have traditionally been able to do. This new ability to co-produce knowledge is foundational to all the processes described here.

The term crowdsourcing was coined in 2006 in *Wired* magazine to describe an extension of outsourcing (Howe 2006). It was viewed as a novel way in which corporations could use the Internet to access large pools of (often untrained) international labor to complete menial tasks cheaply (Ettlinger 2016). The process was quickly adopted by proponents of open content and collaboration, who have argued that crowdsourcing has the potential to empower regular citizens to produce knowledge and products that disrupt proprietary business models (e.g., Benkler 2006, Lievrouw 2011). Crowdsourcing is largely thought to function via Linus’s Law, which says that all problems can be fixed quickly given enough participants in a process. Wikipedia is perhaps the most widely-cited example of open knowledge production through crowdsourcing, but other examples abound (Elwood et al. 2013). The data resulting from crowdsourcing are sometimes referred to as user-generated content (UGC), which is generally placed in contrast to professionally-generated content (Cooper et al. 2017). Importantly, though, some of the neoliberal elements of crowdsourcing’s inception remain, and scholars have criticized how even well-intentioned crowdsourcing efforts can responsibilize citizens to collect their own data and then exploit that data for profit (e.g., Leszczynski 2013). This underscores the colonial potential of crowdsourcing, as highlighted in the introduction.

VGI is regularly framed as a subset of crowdsourcing, although the terms do not perfectly overlap (Cooper et al. 2017). This term was first introduced by geographer Michael Goodchild (2008) to refer to the ways in which citizens can now use GPS units (often embedded in mobile devices) to act as ‘voluntary sensors’ to collectively produce geospatial intelligence. Goodchild’s piece set off a series of discussions within the discipline about how the term should be defined, including debates of whether information is actually being produced voluntarily (e.g., Harvey 2013; van Exel et al. 2011), whether there is a strict binary between VGI and professional geospatial data (e.g., Cinnamon 2015), the relationship between VGI and hacking (e.g., McConchie 2015), and others. Despite these debates, there is widespread agreement that VGI is both a product of and has contributed to broader shifts in how society thinks about and engages with geospatial data. Within the discipline of geography, these shifts have been encapsulated by the term ‘neogeography’, which broadly refers to the opening up of geospatial knowledge production to new individuals and methods (Capineri 2016; Graham 2010). While VGI and other neogeographic practices are not intrinsically digital in nature, they most often do result in digital data production thereby expanding the so-called geospatial web, or geoweb (Elwood 2008; Elwood et al. 2013). Taken together, these processes represent a fundamental shift in who is actively participating in the negotiation of geospatial knowledge, data, and representations, and therefore shifts in how geospatial data are productive of power relations. For some these shifts hold great potential for the radical democratization of the epistemological foundations of cartography (e.g., Young and Gilmore 2017), while for others VGI signals the production of new data inequalities and tyrannies (e.g., Elwood et al. 2013).

Debates around the democratizing potential of VGI are perhaps nowhere more relevant than in the Global South, given the historical use of crowdsourcing to exploit international divisions of labor as well as existing digital and data divide. Researchers, international organizations, and corporations have all explored various approaches to bringing the benefits of VGI to the Global South. Perhaps the earliest and largest set of academic research has focused on how VGI can be used for crisis or disaster management within international settings (Goodchild and Glennon 2010). Zook et al. (2010), for example, described how VGI was used to support responses to the 2010 earthquake in Haiti. Geoweb applications including OpenStreetMaps (OSM) and GeoCommons were set up to allow users from around the world to produce maps that could be used to direct the emergency management practices of international organizations that were in the field. OSM users, for instance, used satellite imagery to trace out information about how streets, buildings, and other infrastructure were impacted by the earthquake. Other applications, like Ushahidi, allowed survivors to use SMS, MMS, or online interfaces to send messages directly to emergency responders (Zook et al. 2010). This allowed survivors of the earthquake to text for help, and for emergency response units to quickly and efficiently locate and respond to incidents. These platforms, and others, have also been used to respond to a range of natural and humanitarian crises, including earthquakes, election tampering, refugee crises, the spread of epidemics, and more (Weyer et al. 2019; Zambrano 2014). Even within this relatively well-developed area of research, though, many gaps remain. Porto de Albuquerque et al. (2019) argues that there still aren’t sufficient methodological guidelines for how to implement VGI within humanitarian relief, and that more research is particularly necessary in the area of validation.

Although it has been most popularized within disaster management, the method has also spread to other areas of sustainable development. VGI has proven successful for Global South projects in the areas of mapping land cover and agriculture (Fritz et al. 2009; Lesiv et al. 2018; See et al. 2013), citizen science for conservation (Genovese and Roche 2010; Pocock et al. 2018), urban planning (Diaz 2016; Ruiz-Correa et al. 2017), and more. VGI projects have been viewed as having particular potential across Africa, and as a result the UN Economic Commission for Africa (ECA) produced a 2017 guideline document to help organizations implement their own crowdsourced mapping projects. Numerous VGI projects have been carried out across the continent, including Ushahidi-based election monitoring and Map Kibera in Kenya, iCitizens in South Africa, agricultural support in Uganda, Ebola mapping in West Africa, the mapping of refugee shelters in Somalia, natural disaster response in Tanzania and Malawi, and much more (UNECA 2017; Yilma 2019; Zambrano 2014).

While these projects have demonstrated the potential for VGI across Africa, many research gaps and challenges remain. First, there is still a paucity of data available within and across many African countries. Many of the projects described above are confined to a few specific topic areas (e.g., disaster management, urban governance, agriculture), relatively small scales (e.g., single communities or countries), and projects of relatively short duration (e.g., a single election cycle). This ensures the continuation of data gaps and inequalities across the continent—especially with regard to data that are difficult to glean from satellite imagery and that come from more rural or remote areas. There also tends to be more experimentation with crowdsourcing in countries with more developed ICT infrastructure and higher GDP, such as Kenya and South Africa. Second, and perhaps more importantly, many of these VGI projects are strongly driven by international NGOs that hold Western views of development and data. Omanga and Mainye (2019), for example, describe their negative experience, as African scholars, participating in a research project that sought to evaluate the effectiveness of Ushahidi at monitoring electoral-related violence in Kenya. They found that the actual users of Ushahidi tended to be networks of international NGO employees, rather than Kenyans—and that this greatly biased the knowledge produced. In their words, they found that the relationship between digital innovation, NGOs, and funding agencies “reproduced a hierarchical, top-down ‘developmental’ logic, whose main inspiration was an uncritical techno-determinist rationality.” (Omanga and Mainye 2019) Their experiences are consistent with broader critiques of how supply-side aid (Fechter and Schwittay 2019) and digital humanitarianism (Burns^2014^) use development to extend Western and neoliberal rationalities. There is a risk that these projects feed back into the processes of colonialism and neoliberalism that VGI would ideally resist.

More work is therefore needed to understand how African organizations agencies might implement and sustain their own comprehensive VGI projects. As Graham et al. (2014) point out, this research needs not only to identify the technical constraints that African organizations face in implementing technology-based projects, but also the social, political, and even regulatory barriers to the success of VGI in particular African contexts. Other important areas of research include what types of digital platforms can be developed to overcome the resource and ICT constraints faced in many African countries (Chaula 2019); how methods can be developed to minimize bias and uncertainty within African VGI datasets (Basiri et al. 2019; Bordogna et al. 2016; Brown 2017); how VGI projects might be made scalable and sustainable (Arora 2016); and what relationship African-driven VGI projects can and should have to broader patterns of neoliberalization and colonialism (Arora 2016). While this paper cannot answer all of these large questions, it attempts to begin the conversation by examining a VGI project designed to help AfLIA, an international NGO based in Ghana, map all public libraries across the continent. Our hope is that, by describing the challenges and benefits that AfLIA and their partners experienced throughout the project, we will provide a road map for other African organizations interested in implementing their own VGI projects. This case study is described in the next section.

## Case study: mapping public libraries across Africa

This paper focuses on a VGI project that has emerged out of a research collaboration between the University of Washington and AfLIA. This project, called Advancing Library Visibility in Africa (ALVA), broadly examines the relationship between public libraries and sustainable development across sub-Saharan Africa. Public libraries and development organizations share many common goals that make them strong potential partners. Both groups seek to build strong community partnerships as they work toward sustainable development goals by increasing access to information and communication technologies (ICTs; Abdulla 1998; Akintunde 2004; Bamgbose and Etim 2015), promoting literacy and lifelong learning (Alabi et al. 2018), providing health and social services (Albright 2007), and much more. Agbo and Ongekweodiri (2014) go so far as to describe libraries as ‘engines of development’ to underscore the powerful and active role that these institutions might play. In spite of these commonalities, libraries are often overlooked as development partners (Fellows et al. 2012). Many librarians argue that this is often the result of a perception problem—libraries are not framing their own work in terms of development, and development organizations therefore do not see the potential value in partnerships. As a result, libraries are not getting the support that they need in order to effectively implement services that will advance local development (Ashraf 2018; Bradley 2016; Moahi 2019). This makes the role of libraries within development more of an unrealized potential than a reality (Moahi 2019).

ALVA responds to this challenge by asking how public libraries can overcome perception issues and fully demonstrate their value as development partners. Long term the project hopes to build strong data culture and data collection expertise within libraries across Africa, so that they can collect, analyze, and present data that documents the impact they have on their local communities. In the short term, however, the team recognized that a much more basic data need was more pressing—in most countries across sub-Saharan Africa, there is a lack of data on even the number of libraries and their locations. The project team therefore determined that collection of geospatial and organizational information (including contact information) for libraries across the continent was the highest priority. This could then serve as a base layer for presenting additional information (e.g., development impact) about libraries in the future.

Because library location data was not officially collected by government agencies in most countries, the project team chose to explore a VGI approach. However, the project team was concerned that a traditional crowdsourcing approach (targeting the general public) would fail given that many libraries are in remote, rural locations with little ICT infrastructure and that the mapping of public libraries seemed unlikely to garner broad public interest. This feeling was confirmed by the low number of public libraries that we found on other crowdsourcing platforms, such as OSM, relative to rough estimates published by the International Federation of Library Associations and Institutions (IFLA; IFLA 2018). We therefore chose to adopt a facilitated VGI approach (Cinnamon and Schuurman 2013), in which we solicited the help of targeted library professionals and library networks to crowdsource library locations. Our plan was to develop a public-facing mapping platform to which anyone could contribute data, but then to train in-country ‘Champions’ within every country across the continent to direct data collection activities within their professional networks.

We first developed a test platform using the Ushahidi platform, which we piloted with a group of 120 participants from one of AfLIA’s library training programs. The platform asked users to identify the location of their own library and then to provide attribute information including its name, what type of library it is, contact details, and what types of services it provides to patrons. The goal of the pilot was to determine the general usability of the platform for users, as well as to understand challenges they faced in contributing accurate geolocation information about their library. By the end of the test 28 unique library sites had been contributed across 11 countries. Users were invited to participate in a follow-up survey which asked them about their experience with the platform. We found that multiple users had difficulty with the auto-location feature of the platform and that some participants became confused by some of the terminology and concepts related to the attribute data we were attempting to collect. Based on feedback we chose to develop a new platform using the ArcGIS Online Crowdsource Reporter application, with a more user-friendly interface and a much shorter survey. Users are now only asked to provide the location of the library, its name, its type (public, academic, etc.), and contact information for both the library and user. They also have the option of submitting a photograph of the library. Eventually we would like to transition the platform to open source software, so that it can be sustainably hosted by AfLIA instead of the University of Washington.

We then began the process of selecting our in-country Champions. The goal was to select individuals that are well-connected within the public library sector of their respective country, so that they would be able to advertise the platform to a wide range of librarians around the country. We began the process with Champions from three countries and then slowly added additional countries to the project. After a Champion was selected they were trained by AfLIA on how to use the crowdsourcing application. At that point they were then in charge of organizing crowdsourcing efforts within their country. They chose to do this in varied ways—some organized training sessions for librarians; some distributed training videos or other material to contacts, but didn’t formally train them within an interactive setting; some simply distributed the link to the site to their contacts; and some chose not to involve other librarians at all, but instead to travel around the country doing the mapping themselves.

We also developed a separate platform, using ArcGIS Online Crowdsource Manager, that allowed the research team to implement a quality control process for library submissions. Once a library is submitted, researchers at AfLIA take several steps to ensure that it seemed like a reasonable location. First, they check the timestamp of the submission to verify that it was submitted during working hours. Throughout the project we found that if a location was submitted outside of these work hours, then the submitter was often not actually at the library location. Since they would often use the application’s autolocation feature, these submissions were often erroneous. Second, using satellite imagery the researchers verify that the submission was on or near a specific building. Third, the researchers compare photographs of the library (either obtained from the submission or found through social media searches) to both satellite imagery of the location and also, when available, Google Street View images. If any of these steps raised questions for the reviewer, then a researcher would reach out to the relevant Champion to get further clarification. The researcher would often share screenshots of satellite imagery or Google Street View images of the submission and ask whether the location seemed to be correct. This would lead either to verification or revision of the location. At the end of this process the library point would be approved. Prior to the stakeholders’ meeting described below, Champions from twenty-three countries were participating in the project.

## Methodology

In October 2019 the research team invited the twenty-three Champions actively involved in the mapping work to participate in a 3-day Champions’ Meeting in Accra, Ghana. The purpose of that meeting was to examine the challenges that the Champions faced in implementing the crowdsourcing process within their country, to discuss opportunities produced by either the mapping process or resulting data, and to brainstorm ways to make the project sustainable in the long-term within their country. Research related to this meeting was divided into two portions. First, Champions were asked to participate in a survey about their mapping work ahead of the meeting. Second, Champions both presented on their mapping progress and also engaged in group work during the meeting itself. The individual and group work presentations were recorded for analysis.

The survey was distributed via email to all twenty-three Champions ahead of the meeting. The email provided them with a link that took them to an online survey portal, where they could choose to take the survey in English, French, Portuguese, or Arabic. Once the Champion chose their language and gave their informed consent to participate, they were then taken to a series of questions that solicited either open-ended or categorical responses. Questions covered the following topics: background on the participant’s job within the library field; their prior experience with performing data collection in their jobs; the bureaucratic, technology, financial, or other challenges that they encountered while participating in our crowdsourcing project; the benefits that they have experienced from participation in the project; and the broader impact they believe that the crowdsourced data could have on their country’s library sector. All twenty-three of the invited Champions successfully completed the survey.

The meeting itself took place over three days and was attended by twenty-two of the invited Champions. Days 1 and 2 of the meeting were a combination of individual presentations to the whole group, question and answer periods with the whole group, and group work in smaller, 5–6 person groups. In most cases the group work resulted in a presentation back to the rest of the Champions, in order to summarize the major conclusions of the group activity or discussion. On Day 1 the participants discussed the following topics: their general experiences with the data collection process; the challenges they encountered; the personal benefits they experienced through participation in the project; a summary of the results from the survey that they took; and an update on the overall progress of the project. On Day 2 they discussed how they think libraries could utilize the project data in their country and how they believe data collection projects could be made sustainable within the country. On the final day the meeting took a slightly different format. In the morning the Champions visited two libraries in Accra, as a bonding experience and form of professional development. In the afternoon the research team then shared preliminary results from Days 1 and 2 with National Librarians from across Africa, who were visiting Accra for the 3rd Meeting of African Library Ministers. This was an opportunity for the team to get greater buy-in from the government agencies that control library activities. As discussed below, greater buy-in from government was a key suggestion from Champions to ensure long-term sustainability for the project.

Analysis for this paper focused on a subset of data from the meeting. First, the meeting began with presentations by the Champions about their experiences with the data collection process. They were asked to design a presentation using a template that focused on the strategies they used to organize crowdsourcing within their country; the challenges they faced in implementing the project, and how they attempted to overcome those challenges; the benefits they’ve experienced through their participation; and any thoughts they have on long-term sustainability of the data collection. These presentations were included in analysis. Second, at the end of Day 1 Champions were asked to perform small group work to identify what they considered to be the top three financial problems, technological problems, bureaucratic problems, and benefits related to the project. Prior to this group work, Champions had given their individual presentations about these topics and the research team had also presented a summary of the survey (which also covered these topics). This group work was used as an opportunity to encourage the Champions to reflect on all of those prior discussions, and to try to come to a consensus around the most important challenges and opportunities related to crowdsourcing. Each group created a presentation summarizing the challenges and benefits that they selected, and these presentations were analyzed for this paper. Third, on Day 2 the small groups were asked to discuss possible strategies for making our data collection process sustainable within their respective countries. The group presentations resulting from those discussions were analyzed for this paper. These three data sets were analyzed alongside the qualitative (open-ended) results of the survey. The researchers performed an inductive analysis of these data using a grounded theoretical approach (Clarke 2003; Glaser 1978; Kitchin and Tate 2000). They analyzed all four data sources together, looking for common trends that shed light on the key challenges, opportunities, and approaches to sustainability related to implementing our crowdsourcing project within participating African countries. This process was iterative and the researchers triangulated codes across the different data sources to ensure consistency (Baxter and Eyles 2010). Results of this analysis are discussed below. Although analysis reflected discussions of both the challenges and benefits of VGI, this paper focuses primarily on challenges in order to highlight the unique barriers that Global South researchers and practitioners need to plan around when designing their own projects. Nevertheless, our Champions saw large benefits to the work, and these are summarized in the conclusion.

## Discussion

By performing the VGI project across twenty-three different countries and giving Champions wide latitude in how they chose to implement it, we allowed for a wide range of experimentation in order to see what techniques most effectively produce crowdsourced data. Despite this flexible approach, our research revealed more similarities than differences across the Champions’ experiences. Although they were coming from countries with different linguistic and cultural histories, library governance systems, economic and development levels, and more, the Champions tended to use very similar methods for contacting librarians and they also faced very similar challenges and opportunities. In fact, toward the end of the meeting one Champion commented that the presentations made them feel much better about their slow progress, precisely because they saw that their struggles were common ones across the continent. Our analysis revealed three take-aways that resonated most strongly across both survey results and meeting discussions—poor Internet connectivity was one of the largest issues faced by Champions; local librarians often resisted participation within the project; and the relationship between the project and existing library (government or non-governmental) organizations presented both challenges and opportunities. In many instances these lessons contradict common assumptions about the advantages and methods of crowdsourcing, and therefore highlight the need to develop unique VGI approaches tailored to the unique context of countries across Africa. Each of these take-aways is discussed below.

### Internet connectivity

One common assumption about crowdsourcing is that it is able to successfully leverage the ubiquity of digital networks to democratize knowledge production. However, our project highlights the deep limits that digital divides continue to impose on crowdsourcing efforts across Africa. Champions overwhelmingly identified Internet connectivity as a challenge that they faced during the project. For some Champions this was a defining feature of all aspects of the project—they live in countries where the Internet is slow, expensive, and regularly disrupted by power outages. These problems made it more difficult for those Champions to contact any librarians, thereby hindering their general progress. In other countries connectivity tends to be more stable but is still affected by unequal infrastructural geographies. Librarians working in remote and rural locations tend to have little access to Internet connectivity, whether through Wi-Fi or mobile data. During the meeting Groups 1, 2, and 3 also shared that some of these rural libraries suffered from lack of access to computers or mobile phones, making Internet access entirely impossible even if the area had some form of connectivity. To some extent this challenge should not be surprising—research on digital divides has long emphasized how uneven access to the material infrastructure of the Web produces asymmetries in what geographies experience digital empowerment (Crampton 2003, 2009; Crutcher and Zook 2009; Elwood et al. 2013; Gilbert 2010; Graham and Zook 2013). These inequalities are also often experienced more dramatically in Global South contexts (Young 2019a, b). Nevertheless, VGI literature still tends to be optimistic about how crowdsourcing opens knowledge production up to even the most marginalized populations. Our research indicates that, without appropriate interventions, crowdsourcing approaches alone will produce deeply incomplete and uneven representations of sub-Saharan Africa. Digital divides produce both inequalities *between* countries and between rural and urban areas *within* countries.

Connectivity challenges were exacerbated by lack of funding and a lack of technical literacy amongst librarians. The project provided Champions with a small stipend to compensate them for the time they spent implementing the project in their countries. However, following a traditional crowdsourcing approach, the project did not provide librarian users with financial support. Our assumption was that submission of a single library location would be a quick and relatively simple process, thereby not justifying financial support. Unfortunately, Champions found that this assumption was not borne out in reality. As one argued in their survey, “Calling and communicating with librarians needed data and airtime and this was very costly especially in my country data is very expensive.” Rural librarians, in particular, often did not have enough money to make phone calls, much less to use data to submit their library location on our site. As one Champion said in their survey, “At times some Librarians are having no credit to access WhatsApp, to complete the data, but willing to call back once they have credit.” In this instance funding only produced delays, since librarians would eventually have data that they could use. In other cases Champions chose to use their own funding to purchase data packages for librarians: “I mostly had to call, which required airtime and in some instances I had to buy data for the participants to connect their phones online.” Notably, these costs weren’t only (or even mostly) associated with the submission of data—they were largely incurred because Champions had to spend a large amount of time on the phone with librarians, walking them through the location submission process. This was because many librarians had very low levels of technical and cartographic literacy. Champions found that many librarians could not use their phones for anything other than phone calls; that some librarians did not know how to use social network applications; and that they had difficulty navigating the platform itself, even when provided with a full explanation of the process. Other librarians also had a great deal of trouble zooming and panning the map to locate their library. Whenever possible we recommended that they use a smartphone so that they could take advantage of its GPS unit. In these instances the librarian could use the autolocate feature on the map, assuming that they were doing the submission while at their library. However, some librarians only had access to computers or older smartphones, and therefore could not use this feature. Throughout the quality control process we regularly found that manual placement was highly inaccurate. We believe that librarians would often not zoom the map sufficiently, and would simply place their library in the general space of their country or city. This would produce very inaccurate locations when a user zoomed into the submission. While our quality control process was designed to reduce some of the inaccuracies, it is certainly imperfect given the lack of ground truthing. Some risk therefore remains that these rural areas are less accurately represented. Given that these literacy issues also tend to be correlated with rural areas, this means that rural areas are not only more likely to be unrepresented within maps but also more likely to be inaccurately represented.

In order to overcome these issues Champions were forced to use a wide variety of methods to support local librarians. This represents a need for a much higher level of support and intervention than is used by most crowdsourcing projects. First, Champions tended to use many different communication methods to ensure that the librarians understood the process—it was not as simple as advertising a URL within their social media networks. These methods included data-based modes of communication (email, social media, WhatsApp, sharing of downloadable videos), phone calls, and in-person meetings. Nearly half of the Champions indicated, in the survey, that they needed to use at least two of these three forms of communication. Five of the twenty-three respondents indicated that they regularly used all three. One Champion explained their communication workflow as the following: “Email sent to libraries explaining the project and its objectives and seeking their collaboration; This is followed by phone calls and further clarifications/explanations on the project; in some cases personal meeting is required on site and assistance provided to submit the data.” Another Champion followed a similar process, but began with WhatsApp and phone calls: “Communication through whatsapp [sic] or phone calls; training for data input on the platform if consensus is reached; traveling to the place of libraries in case of technical difficulties.” We were particularly surprised to see how often the Champions were physically traveling to libraries, since this was not an expectation of the project. Several of the Champions were physically traveling to all library locations and submitting locations themselves. This was facilitated if traveling between the libraries was a regular part of their job. As one Champion said, “Sometimes I take advantage of my routine monitoring trips to collect data and brief staff on the significance of the project.” In these instances the method more resembled an authoritative data collection effort than it did a crowdsourcing project, since an officially trained project representative was going from site to site to collect the data. However, the Champions emphasized that this was often necessary to overcome the ICT access and technical literacy issues faced at some libraries, particularly in rural areas. The take-away here is that a more traditional VGI approach would have been highly biased toward more affluent African countries and urban areas, as compared to our methodology. This underscores the importance of augmenting VGI projects with higher levels of support, in order to overcome the biases produced by digital divides across Africa.

### Librarian motivation

Crowdsourcing projects also require a large and motivated public in order to succeed. Unfortunately, Champions found that there was not a sufficient volunteer or open data culture within this library community to easily sustain crowdsourcing—a second important challenge was motivating public librarians to participate in the project. This challenge is concerning because librarians should, hypothetically, be ideal participants in a project intended to benefit the public library sector. Brown (2017) argues that a focus on the technological components of VGI projects is often misplaced—the most important aspect of these projects lies in understanding how to isolate and motivate a particular public to produce high quality data. This is easiest when the mapping work is closely aligned to the livelihoods or everyday needs of those being asked to engage in mapping (Brown 2017; Chuene and Mtsweni 2015). In the context of this project, the best possible users should be librarians and library users. However, our Champions found that many librarians approached the project with a great deal of skepticism and resistance. In some cases, it was unclear why the librarians didn’t wish to participate—they were often willing to learn about the project and even undergo training, but they wouldn’t follow through with data submission. One Champion said, “One main issue is that of getting Librarians to participate in the project. They are willing to go through the training on filling out the form but for some reasons they don’t end up filling it.” Champions were often quite persistent in following up in these cases, and would sometimes finally get a librarian to submit data. However, if that data appeared inaccurate during our quality control process, they then wouldn’t get any responses from the librarian for revision. As another Champion said, “Reluctance of some libraries [is a problem]; some libraries promise to fill in the form, but they don’t even after several follow up calls are made. Others take long to make the correction requested for, some don’t even bother.” In some cases Champions felt they would need to travel to these libraries and do the mapping work themselves, due to total lack of response or engagement from the librarians.

In other instances, the Champions had a clearer idea of why librarians chose not to engage in the project. Some librarians did not believe that the project would directly benefit them at a personal level, and therefore did not see a point to their participation. Champions were trained to describe how the data could be used for advocacy purposes, to increase the visibility of libraries in the eyes of potential funders. However, this purpose was often not concrete enough. As one Champion said, the librarians often did not want to participate in data collection, “especially because they did not understand the immediate benefit to the library.” Instead, this same Champion said, these librarians “requested to know if AfLIA had an intervention plan to support them. When asked to give an indication of their requirements, they stressed donation of books, and computers as well as training in use of ICTs, etc.” Others wanted direct, personal benefits instead of benefits to the library sector. One Champion stated that the “main challenge was getting some librarians to submit data. They were eager to know how the project would benefit them individually not as an organization.” Others described how “librarians and library sector leaders seem to cooperate for projects that pay them for volunteering” and “there is always high expectation for financial rewards the moment you try to engage other people”. In preparing their summary of the bureaucratic changes they faced, Group 2 tied this dynamic back to the practices of NGOs in their countries. They argued that “NGOs are paying for research data and librarians are expecting Champions to also pay them.” Group 1 listed ‘motivation costs’ as a financial challenge faced by the project. It seemed clear in each of these cases that librarians did not want the abstract benefits often offered as motivation by crowdsourcing benefits, but instead expected direct payment for their labor.

Other librarians feared using the Internet or sharing data. Fear of the Internet, or what several Champions referred to as ‘technophobia’, often seemed to be related to low levels of technical literacy. As one Champion argued, “some librarians are having fear on Internet, hence they assume it is complicated to do online input, especially the GPS, to locate the Library.” Others seem to have a nebulous fear of sharing information about their libraries, perhaps because the information might be used against them (e.g., if data collection strategies are used by governments to restrict funding based on performance). One Champion said that library authorities would not “grant permission to library staff to provide the data on the grounds that such data are confidential and are meant for internal use only”. Another Champion even indicated that they faced “lack of data accessibility in certain areas linked to insecurity in these areas due to terrorism.” In most instances these reasons were not well explained or developed, since they are only based on the perceptions and assumptions of Champions (who often had low levels of access to the librarians themselves). More research is therefore needed to really understand the motivations of potential crowdsourcing participants in these contexts. The key take-away, though, is that VGI projects in these countries face large hurdles in getting widespread buy-in from the public. Given that VGI projects tend to be *most* successful with those individuals that most directly interact with the locations being crowdsourced on an everyday basis (Brown 2017), it is worrisome that librarians are so highly resistant to participation. This is particularly the case given that they are receiving high levels of support and encouragement from Champions, which doesn’t usually happen in more passive forms of crowdsourcing.

This isn’t to say that the Champions were unsuccessful at eventually motivating many of the participants—they used a variety of tactics to overcome this barrier. As discussed in the next section, many Champions found that they could motivate librarians by leveraging their relationships with AfLIA or with national library authorities. For example, Champions requested a letter from AfLIA detailing the organization’s support for the project and the benefits of participating. Champions believed that this letter greatly improved their success in soliciting participation. Other Champions obtained similar letters from their own National Librarian, to indicate that in-country authorities were supportive of the project. In other instances Champions were able to successfully motivate librarians by sharing some of the more abstract or indirect benefits of participation, including increasing the visibility of their library, expanding connections to others in their country’s library field, and increasing their technological capacity through training to use the platform. Successful approaches for motivation varied by country and even local library, and at times no approach was successful at all. More research is therefore needed to understand the cultural dimensions of VGI participation. VGI projects need to consider how local cultural understandings of payment/volunteering, data, and privacy intersect with motivations to engage in crowdsourcing, and either adapt their project (e.g., provide payment) or work to change that particular culture (e.g., develop non-payment based motivation strategies grounded in the local culture). Other aspects of our project have begun to explore how cultural understandings of knowledge production impact local data culture (Lynch et al. 2020), but much more research is needed in this area.

### Relationships to existing library authorities

Finally, crowdsourcing projects are often viewed as a challenge to or shift away from authoritative forms of data collection. However, our Champions largely viewed it as the opposite—as a method for increasing the involvement of authorities in data collection. Negotiating the relationship between this project and existing library authorities represented both a challenge and an opportunity for all of the Champions. Like the other challenges, this shouldn’t be a huge surprise—all forms of neogeography represent a unique challenge to authoritative forms of data collection, and therefore must negotiate their relationship to authorities. What was surprising was the strength of the desire by both Champions and librarians alike to ultimately fold the project into traditional library hierarchies. A common theme amongst Champions was the need to get authorization for the project from government authorities (e.g., a secretary of general within the government ministry that has authority over public libraries). Approximately half of the Champions needed this authorization so that they could engage in project activities themselves. More importantly, though, the librarians themselves would expect to see that permission was expressly granted before they were willing to participate in the project themselves, in the form of the letters discussed in the last section. One Champion told us, for example, that they encountered “reluctance of library staff to provide data unless permission is granted by employer in a very formal way”. At the meeting, Group 4 presented that they felt that these hierarchies were sometimes used as an excuse to justify non-participation. In these cases lack of authorization wasn’t actually an issue being faced by the participant, but was a fabricated excuse used to cover up whatever the librarian’s real motivation was for not participating. They could then displace blame for their lack of participation onto someone higher up in the hierarchy. Interestingly, the members of Group 3 found that they did not encounter many of these bureaucratic hurdles. They attributed this to the fact that many of them already hold very high positions within their own library sector, and therefore already have the authority to take on any project that they might like. They argued that this provides a strong justification for more formally integrating the project into regular government operations, rather than continuing to frame it as VGI.

Interestingly, while inclusion of the project within existing organizational structures was extremely important for political and symbolic reasons, it did not provide Champions with many additional resources. In particular, most Champions reported that their governments did not have the resources to connect the Champions with librarians that might want to participate in the project. Champions reported that there was no existing database of libraries nor “existing formal and informal communication networks” for networking with librarians. However, Champions saw this as a key opportunity for the project—it gave them the justification to create these networks. Several Champions reported that they had created their own WhatsApp chat group for public libraries for this project, and they were now using it to talk about other opportunities such as “Library event happenings, studying opportunities, [and] funding opportunities.” Others reported opportunities for building mentorship opportunities with younger librarians. In the long run they believed that these networks, alongside the data they were collecting, would also be valuable for other training and advocacy activities.

In the end every single group at the meeting recommended increased integration between the project and their respective national governments. The official recommendation that they put forward was that the project’s data collection activities should be integrated into the mandate of each country’s National Library system, with implementation delegated to an appropriate subdivision. They viewed this as a far more sustainable and effective method of maintaining up-to-date information about library locations in their countries. The take-away here is that the momentum of the project is toward more organizational integration, not less. In this case crowdsourcing has been viewed not as an attempt to privatize data collection practices that have historically been carried out by the government, but instead as a method for government agencies to start collecting particular types of data. Thus far this approach has seemed effective—when recommendations from the Champions were later presented to national library directors and ministers, these government actors seemed (1) to support the activities and (2) to be eager to ensure that their country was keeping up with data collection in relation to other countries. As the project continues to negotiate the relationship between VGI and government, it will draw on the experiences of other governments that have leveraged crowdsourcing methods (see, e.g., Haklay et al. 2014).

## Conclusion

VGI projects like OSM, crisis mapping in Haiti, and Map Kibera have effectively captured the academic imagination, highlighting the potential power of crowdsourcing as a tool for knowledge production in the Global South. However, their success has also normalized a particular vision of what a Global South crowdsourcing project might look like—focused on topics of high interest to the global development community; on features that are easily verified by remote sensing; on locations with higher ICT penetration, affluence, or NGO presence; etc. As Omanga and Mainye (2019) point out, these common models of VGI can undermine the method’s empowering potential by allowing organizations from the Global North to largely drive what types of knowledges are created through crowdsourcing. In this sense, maps produced through VGI risk, drawing on Spivak (1999), acting as tools through which the Global North continues to ventriloquize the Global South. This, in turn, can turn VGI into a tool for data colonialism rather than democratization (Dé et al. 2018; Fraser 2019; Thatcher et al. 2016; Young 2019a).

This paper asks what a VGI project might look like if it is co-created and co-implemented with African organizations, with the goal of sustainably handing the project over to those organizations over the long run. The focus of the mapping, libraries, is quite different from many of the features commonly mapped through VGI—libraries are largely ignored by development organizations (Fellows et al. 2012), are located in geographies that have little access to ICT infrastructure and little contact with outside organizations, and are difficult to identify through the use of satellite imagery. We have found that crowdsourcing has been a relatively effective method for mapping libraries and for getting library organizations (and government actors) excited about developing more of a data culture around libraries. However, we also found that crowdsourcing in these contexts has faced different challenges and realized different benefits from other similar projects. The project highlighted how dramatically digital divides can bias crowdsourcing results, as well as the degree to which local cultural views influence public motivations to participate in crowdsourcing. Perhaps most importantly, the project showed how crowdsourcing is viewed by some as a way to influence, and even increase the involvement of, government authorities in mapping instead of as a way to privatize existing government data collection efforts.

All of these findings offers lessons for researchers attempting to implement crowdsourcing projects in the Global South. These projects must be carefully designed so that they account for digital divides, local cultural views of volunteerism and open data, and orientations toward government or organizational hierarchy. We would argue that this is best done through consultation and partnership with partners in the Global South, to ensure that the end result is something that reflects their needs and can be sustained. Naturally, more research is also needed in many of these areas, to ensure greater success. What devices, platforms, and training approaches are most successful at reaching remote and rural communities, to ensure their representation within crowdsourcing projects? Low-data usage platforms like WhatsApp are extremely popular in many of these areas, for example, but have not been fully explored in the context of crowdsourcing. What are local understandings of (open) data across different areas of the Global South, and how do they intersect with public motivations to participate in crowdsourcing? How are ICTs creating or transforming data culture, and how does this produce new democratizing or colonial geographies? What is the relationship between crowdsourcing methods and governments that have not historically collected large amounts of authoritative data? These questions, and many others, must be answered before we really understand the implications of these digital data-production methods across many geographies of the Global South.

While this paper focused on the challenges of implementing VGI projects in the Global South, it is worth noting that the stakeholder meeting also highlighted a range of benefits. Benefits discussed by the Champions included increasing the visibility of libraries, expanding connections between participants and others in the library field, and increasing the capacity of librarians. The first benefit, increasing library visibility, is a direct result of having location data about libraries, and is a common benefit of all crowdsourcing projects. The other two benefits are more indirect forms of empowerment that are not directly related to the collected data, but instead to participation in data collection itself (see, e.g., Elwood 2002; Young and Gilmore 2013). Champions argued that the project forced them to create new communication channels (e.g., via WhatsApp) with their colleagues across the country, led them to learn more about the services of other libraries, and more. They believe that this networking will spur future cooperative efforts across the field. They also found that participation in the project expanded the data and technology skills of themselves and participating librarians. One Champion found that the participating “librarians improved their skills and some were excited to be part of a global research [project].” They argue that these skills would broadly advance their own professional lives. In the end the Champions unanimously agreed that the project’s benefits outweighed the challenges, and that it was vital to ensure the project’s long-term sustainability. This reflects a strong belief that it is worth navigating difficult challenges to expand data collection and culture in the Global South.

ALVA will continue to explore many of these questions as it continues to expand to additional countries. Since the completion of the Champions’ meeting data collection has expanded to an additional seven countries. Just over 700 libraries have been submitted to the site and approved, and an additional 200 locations are proceeding through the quality control process. In the long run the project will explore what additional types of information it can collect about the libraries that have been mapped, with the ultimate goal of building a powerful platform that libraries can use to collect, analyze, and present data that documents the impact they have on their local communities. Our hope is that this will make libraries more visible as local partners and champions of community-driven development. Along the way we expect to uncover many other lessons about data culture and knowledge production across the Global South.

## Data Availability

This dataset includes sensitive human subjects information, which is currently not available for public consumption.
